# Micro adénome à prolactine à l’âge de la ménopause

**DOI:** 10.11604/pamj.2017.27.177.11677

**Published:** 2017-07-05

**Authors:** Ines Barka, Emna Dendana, Nesrine Chikhrouhou, Amel Maroufi, Maha Kacem, Molka Chadli, Koussay Ach

**Affiliations:** 1 Service d’Endocrinologie, CHU Farhat Hached de Sousse, Tunisie

**Keywords:** Prolactinome, péri ménopause, agoniste dopaminergique, Prolactinoma, perimenopause, dopaminergic agonist

## Abstract

L'adénome à prolactine est rare chez la femme âgée. Le tableau clinique peut être confondu avec les manifestations de la ménopause, rendant son diagnostic parfois difficile. Nous rapportons une observation sur les particularités d'un micro adénome à prolactine survenant chez une femme âgée de 57 ans, qui a présenté une aménorrhée secondaire sans bouffées de chaleur associée à une galactorrhée évoluant depuis 2 ans. L’examen physique confirme la galactorrhée et la biologie montre une hyperprolactinémie à 2735 mUI /L, FSH = 15,1 UI/L et LH = 4,1UI/L. L’IRM hypophysaire montre un adénome gauche de 8mm. L'évolution sous traitement dopaminergique était marqué par la reprise transitoire des cycles et apparition de bouffées de chaleur, normalisation de la prolactinémie et diminution de la taille de l'adénome.

## Abstract (en)

Prolactin-secreting adenoma is rare in elderly women. Patient’s clinical picture may be confused with that of menopause, making diagnosis sometimes difficult. We report the case of a 57-year old woman with a 2-year history of secondary amenorrhea without hot flushes associated with galactorrhea in order to highlight the peculiarities of prolactin-secreting microadenomas. Physical examination confirmed the diagnosis of galactorrhoea and biology showed hyperprolactinemia at mIU/L, FSH = 15.1 IU/L and LH = 4,1 IU/L. Pituitary MRI showed left adenoma measuring 8 mm. Patient’s evolution under dopaminergic treatment was marked by the recovery, for a transitional period, of mestrual cycles and the occurrence of hot flushes, normalization of prolactin levels and reduction of adenoma size.

## Introduction

L’adénome à prolactine est l'apanage de la femme jeune entre 20 et 40 ans [1, 2], il est rarement diagnostiqué à l'âge pré pubertaire et au cours de la ménopause [3]. En effet, les prolactinomes sont rares chez la femme ménopausée [4]. Le tableau clinique de l'hyperprolactinémie peut être confondu avec les signes de la ménopause rendant son diagnostic parfois difficile. Le traitement dopaminergique n'est pas systématique. Il doit tenir compte du risque de fragilisation osseuse et du risque de néoplasie qui conditionnent sa prise en charge ultérieure [5, 6]. Nous rapportons une observation sur les particularités de l'adénome à prolactine survenant chez une femme à l'âge de la ménopause.

## Patient et observation

**Figure 1:**
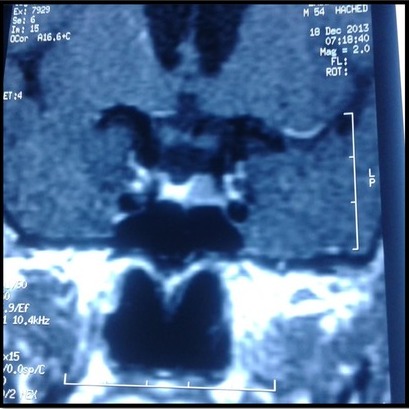
IRM hypophysaire de la patiente avant traitement: micro-adénome gauche de 8 mm

**Figure 2:**
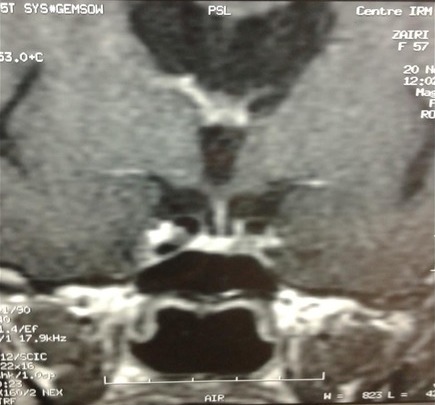
IRM hypophysaire de la patiente après traitement dopaminergique: régression de la taille du micro-adénome (5 mm)

La patiente W.Z âgée de 57 ans consulte pour galactorrhée associée à une aménorrhée secondaire sans bouffées de chaleur évoluant depuis 2 ans. Dans ses antécédents familiaux, on note un diabète de type 2, elle n'avait pas d'histoire de stérilité. Elle avait mené 5 grossesses dont la dernière remontait à 17 ans. A l'anamnèse, on trouve la notion de prise de psychotropes pour des cervicalgies pendant une année: amitriptyline 25mg/j et sulpiride 100mg/jour. La symptomatologie a persisté même après l’arrêt du traitement pendant deux ans. Par ailleurs, elle ne rapportait ni céphalées ni troubles visuels. L’examen physique de la patiente montre: poids: 93 kg avec un indice de masse corporelle à 32 kg/m^2^; tour de taille: 90 cm; tension artérielle: 130/80 mm Hg; pouls à 85 cycles par minute; thyroïde non palpable; galactorrhée bilatérale; absence de syndrome dysmorphique. Son exploration biologique montre: glycémie à jeun: 5,99 mmol/l, glycémie post prandiale à 8,08 mmol/l, créatinine sanguine: 43µmol/l, LDL cholestérol: 4,77 mmol/l. L’exploration hormonale a objectivé une hyperprolactinémie à 91 ng/ml (2735 mUI/L), avec une T4 libre à 10,3 pg/ml et une TSH à 0,88m mUI/L. Le taux d’œstradiol était à 50pg/ml, la FSH et la LH étaient respectivement à 15,1 UI/L et 4,1 UI/L. L’IRM hypophysaire a mis en évidence un micro adénome gauche de 8mm de diamètre (Figure 1). L’écho-mammographie a montré une papillomatose diffuse bilatérale. L’osteodensitométrie au site vertébral a montré un T score à-1,3. La patiente a été traitée par cabergoline 0,5 mg par semaine pendant 12 mois. L’évolution était marquée par la reprise transitoire des cycles suivie d'une aménorrhée avec apparition de bouffées de chaleur, et par la normalisation de la prolactinémie: 8ng/ml et augmentation du taux des gonadotrophines (FSH: 84 UI/l, LH: 23 UI/l). A l’IRM hypophysaire, on a noté une diminution de la taille de l'adénome à 5 mm (Figure 2).

## Discussion

Nous avons rapporté l'observation d'un micro adénome survenant chez une femme en périménopause révélé par un syndrome d'aménorrhée galactorrhée. L'hyperprolactinémie était associée à une ostéopénie et une papillomatose mammaire. L'évolution sous traitement dopaminergique était marquée par la normalisation de la prolactinémie et la diminution de la taille de l'adénome. La prévalence globale des adénomes à prolactine est estimée à 0,66/1000 habitants. Il touche la femme jeune entre 20 et 50 ans [7]. La distribution selon le sexe de ces adénomes varie en fonction de l’âge. En effet, avant l’âge de 60 ans, le sexe ratio est égal à 10/1 alors qu’au-delà de 60 ans, la fréquence devient égale entre les 2 sexes [2]. Chez les femmes ménopausées, le prolactinome est rare [4]. L'âge moyen au moment du diagnostic varie de 50 ans à 63,6 ± 7,1 ans [4, 8]. Cette rareté peut être expliquée par le fait qu’ils restent méconnus devant la similitude du tableau clinique avec celui de la ménopause [4]. Chez notre patiente la prise de psychotropes pourrait expliquer l'élévation de la prolactinémie, d'où la baisse des gonadotrophines et l'absence de bouffées de chaleur. Cette hypothèse a été infirmée devant la persistance de la galactorrhée après arrêt du traitement psychotrope. D'autre part, l'hyperprolactinémie a entrainé une suppression des bouffées de chaleur chez notre patiente, ceci a été signalé par les 2 observations rapportées par Bert scoccia [9]. Ceci peut être dû à un excès relatif en dopamine au niveau hypothalamique [10] avec une diminution du rapport norépinephrine/dopamine en cas d'hyperprolactinémie. Il y aurait également, une diminution de la stimulation du centre de thermorégulation, qui est anatomiquement proche des neurones hypothalamiques à GnRH [11]. Par ailleurs, l'évolution de l’hyperprolactinémie à l’âge de la ménopause comporte des risques. Le premier risque concerne celui de l'ostéoporose. Ce risque a été signalé par Mazziotti qui a montré que les femmes ménopausées présentant un prolactinome ont un risque significativement plus élevé de fractures vertébrale sen comparaison avec les femmes ménopausées sans hyperprolactinémie de même âge [5]. C'est un effet indépendant de la fonction gonadique. Il serait dû à l'action directe de la prolactine sur le remodelage osseux. En effet, elle entraine une diminution de la minéralisation osseuse et une augmentation de l’expression de RANKL/ ostéoprotégérine, la résultante serait alors une accélération de la résorption osseuse [12]. Ce risque semble être probable chez notre patiente puisqu’elle a déjà une ostépenie vertébrale en péri ménopause. Par ailleurs, l'hyperprolactinémie chez la femme ménopausée présente un risque de développement de cancer du sein Selon le travail prospectif de Tworoger, le taux de prolactine plasmatique antérieur est considéré comme un marqueur de risque du développement du cancer du sein après 10 ans d'évolution chez les femmes ménopauses surtout en cas de positivité des récepteurs hormonaux et en présence de métastases [6]. En fait, la prolactine augmente la prolifération et la motricité cellulaire qui sont deux processus importants dans les derniers stades de développement tumoral [13]. Ce risque doit être pris en considération chez notre patiente d'autant plus qu'elle a présenté une papillomatose bilatérale diffuse, considérée comme une lésion pré néoplasique. Enfin, le dernier risque concerne le risque cardio-métabolique inhérent à l'hyperprolactinémie, qui s'associe à une prise de poids et à une obésité par inhibition du tonus dopaminergique responsable d'une insulinorésistance chez des femmes non obèses [14]. Néanmoins, ces études n'ont pas inclus des femmes ménopausées. L’évolution naturelle des micro-adénomes à prolactine semble être stable. La plupart des prolactinomes de petite taille restent au stade de micro adénomes sans évolution au cours de la vie. Les études autopsiques retrouvent un micro-adénome dans 12% des cas [15]. Pour ces raisons, le traitement médical est controversé [16, 17]. Il est indiqué lorsque les facteurs de mauvais pronostic sont présents et fait appel aux dopaminergiques comme c’est le cas chez notre patiente. L'efficacité du traitement a été attestée par la normalisation de la prolactine et la reprise transitoire des cycles. De plus, nous avons noté une ascension des taux des gonadotrophines correspondant aux taux de la ménopause avec apparition de bouffées de chaleur.

## Conclusion

Notre observation souligne le fait qu'un micro adénome à prolactine peut s'installer au cours de la péri ménopause entrainant des problèmes de diagnostic différentiel avec la ménopause et la prise de psychotropes. Le micro adénome à prolactine peut être responsable de l'abaissement du taux des gonadotrophines. Le traitement dopaminergique controversé selon la littérature semble être indiqué chez notre patiente devant l'ostéopénie et la papillomatose mammaire. Cette option thérapeutique devrait être confrontée aux données évolutives de ces paramètres sous traitement dopaminergique.

## Conflits d’intérêts

Les auteurs ne déclarent aucun conflit d'intérêt.

## References

[1] Gillam Mary P, Molitch Mark E, Lombardi Gaetano, Colao Annamaria (2006). Advances in the treatment of prolactinomas. Endocr Rev..

[2] Mindermann Thomas, Wilson Charles B (1994). Age-related and gender-related occurrence of pituitary adenomas. Clin Endocrinol (Oxf)..

[3] Fideleff HL, Boquete HR, Suárez MG, Azaretzky M (2009). Prolactinoma in children and adolescents. Horm Res..

[4] Shimon Ilan, Bronstein Marcello D, Shapiro Jonathan, Tsvetov Gloria, Benbassat Carlos, Barkan Ariel (2014). Women with prolactinomas presented at the postmenopausal period. Endocrine..

[5] Mazziotti G, Mancini T, Mormando M, De Menis E, Bianchi A, Doga M, Porcelli T, Vescovi PP, De Marinis L, Giustina A (2011). High prevalence of radiological vertebral fractures in women with prolactin-secreting pituitary adenomas. Pituitary..

[6] Tworoger SS, Eliassen AH, Zhang X, Qian J, Sluss PM, Rosner BA, Hankinson SE (2013). A 20-year prospective study of plasma prolactin as a risk marker of breast cancer development. Cancer Res..

[7] Daly AF, Rixhon M, Adam C, Dempegioti A, Tichomirowa MA, Beckers A (2006). High prevalence of pituitary adenomas: a cross-sectional study in the province of Liege, Belgium. J Clin Endocrinol Metab..

[8] Vroonen L, Jaffrain-Rea ML, Petrossians P, Tamagno G, Chanson P, Vilar L (2012). Prolactinomas resistant to standard doses of cabergoline: a multicenter study of 92 patients. Eur J Endocrinol..

[9] Scoccia B, Schneider AB, Marut EL, Scommegna A (1988). Pathological hyperprolactinemia suppresses hot flashes in menopausal women. J Clin Endocrinol Metab..

[10] Cramer OM, Parker CR, Porter JC (1979). Secretion of dopamine into hypophysial portal blood by rats bearing prolactin-secreting tumors or ectopic pituitary glands. Endocrinology..

[11] Meldrum DR, Erlik Y, Lu JK, Judd HL (1981). Objectively recorded hot flushes in patients with pituitary insufficiency.J Clin Endocrinol Metab..

[12] Seriwatanachai D, Krishnamra N, van Leeuwen JP (2009). Evidence for direct effects of prolactin on human osteoblasts: Inhibition of cell growth and mineralization. J Cell Biochem..

[13] Touraine P (2001). Implication de la prolactine dans le cancer du sein. Médecine Thérapeutique Endocrinol..

[14] Greenman Y, Tordjman K, Stern N (1998). Increased body weight associated with prolactin secreting pituitary adenomas: weight loss with normalization of prolactin levels. Clin Endocrinol (Oxf)..

[15] Buurman H, Saeger W (2006). Subclinical adenomas in postmortem pituitaries: classification and correlations to clinical data. Eur J Endocrinol..

[16] Faje AT, Klibanski A (2015). The treatment of hyperprolactinemia in postmenopausal women with prolactin-secreting microadenomas: Cons. Endocrine..

[17] Iacovazzo D, De Marinis L (2015). Treatment of hyperprolactinemia in post-menopausal women: pros. Endocrine..

